# Single-cell transcriptome analysis of CAR T-cell products reveals subpopulations, stimulation, and exhaustion signatures

**DOI:** 10.1080/2162402X.2020.1866287

**Published:** 2021-01-06

**Authors:** Xiaonan Wang, Carlotta Peticone, Ekaterini Kotsopoulou, Berthold Göttgens, Fernando J Calero-Nieto

**Affiliations:** a Wellcome and MRC Cambridge Stem Cell Institute and University of Cambridge Department of Haematology, Jeffrey Cheah Biomedical Centre, Cambridge, UK; b School of Public Health, Shanghai Jiao Tong University, School of Medicine, China; c Process Development, Autolus Ltd, London, UK

**Keywords:** T cell activation, chimeric antigen receptor, single-cell transcriptomics, CAR-T exhaustion

## Abstract

Chimeric antigen receptor (CAR) T-cell adoptive therapy is set to transform the treatment of a rapidly expanding range of malignancies. Although the activation process of normal T cells is well characterized, comparatively little is known about the activation of cells via the CAR. Here we have used flow cytometry together with single-cell transcriptome profiling to characterize the starting material (peripheral blood mononuclear cells) and CAR therapeutic products of 3 healthy donors in the presence and absence of antigen-specific stimulation. Analysis of 53,191 single-cell transcriptomes showed APRIL-based CAR products to contain several subpopulations of cells, with cellular composition reproducible from donor to donor, and all major cellular subsets compatible with CAR expression. Only 50% of CAR-expressing cells displayed transcriptional changes upon CAR-specific antigen exposure. The resulting molecular signature for CAR T-cell activation provides a rich resource for future dissection of underlying mechanisms. Targeted data interrogation also revealed that a small proportion of antigen-responding CAR-expressing cells displayed an exhaustion signature, with both known markers and genes not previously associated with T-cell exhaustion. Comprehensive single-cell transcriptomic analysis thus represents a powerful way to guide the assessment and optimization of clinical-grade CAR-T-cells, and inform future research into the underlying molecular processes.

## Introduction

The immune system plays an important role in cancer development and treatment, in both solid tumors and hematological malignancies. Multiple approaches have been explored to direct immune cells specifically against cancer cells. Increased attention has been focused on direct manipulation of the patient’s own immune cells through either small molecules or cell therapy,^1^ including the transduction of peripheral blood T-cells of the patient with a chimeric antigen receptor (CAR) directed against an antigen present in the cancer cells. For these treatment protocols, T-cells harboring the CAR (CAR T-cells) are commonly expanded *in vitro* before reintroduction into the patient.

Following highly encouraging clinical trial results, CAR products have already been approved for therapeutic use and many more are at advanced stages of clinical trials.^2,3^ However, relatively little is known about how CARs function from a molecular point of view, especially with respect to their influence on the overall cellular state of the CAR T-cell products. In particular, the cellular heterogeneity of CAR T-cell products remains poorly defined not only in terms of cellular heterogeneity as a result of culture conditions,^4^ but also because not all cells harbor the CAR as well as difficulties associated with recovery and analysis of the cells upon antigen encounter.

Traditionally, transcriptomic studies of the immune system have relied on flow cytometry to obtain large numbers of relatively homogenous cell populations. The more recent adaptation of single-cell transcriptomic analysis has revealed that almost all cell populations thought to be largely homogeneous are in fact composed of clearly identifiable subpopulations.^5^ Technological advances in single-cell RNA-Seq (scRNA-Seq) permit the cost-efficient processing of thousands of cells,^6^ whereas previously this type of analysis was low-throughput and cost prohibitive. Single cell transcriptome profiling also provides powerful opportunities to analyze molecularly the response and behavior of individual immune cells following stimulation.

Here we have performed a large-scale single-cell transcriptomic analysis of CAR T-cells containing a previously described third-generation CAR based on an “A Proliferation-Inducing ligand” (APRIL) that specifically recognizes the B cell maturation antigen (BCMA) and cyclophilin ligand interactor (TACI), both present in multiple myeloma (MM) cells.^7^ We have combined conventional flow cytometry analysis with state-of-the-art scRNA-Seq to characterize in detail three crucial stages of the CAR T-cell production process, namely the starting leukapheresis sample, the *in vitro* generated CAR-T product, and the product upon specific antigen stimulation. Sampling these three stages from three different donors provided the transcriptional profiles of 53,191 cells in total, demonstrated the robustness of the procedure with respect to sample variation, and allowed us to determine molecular signatures associated with CAR activation as well as the small subset of cells displaying an exhaustion signature.

## Results

### A sampling strategy to capture key stages of CAR product development

**Figure 1.**
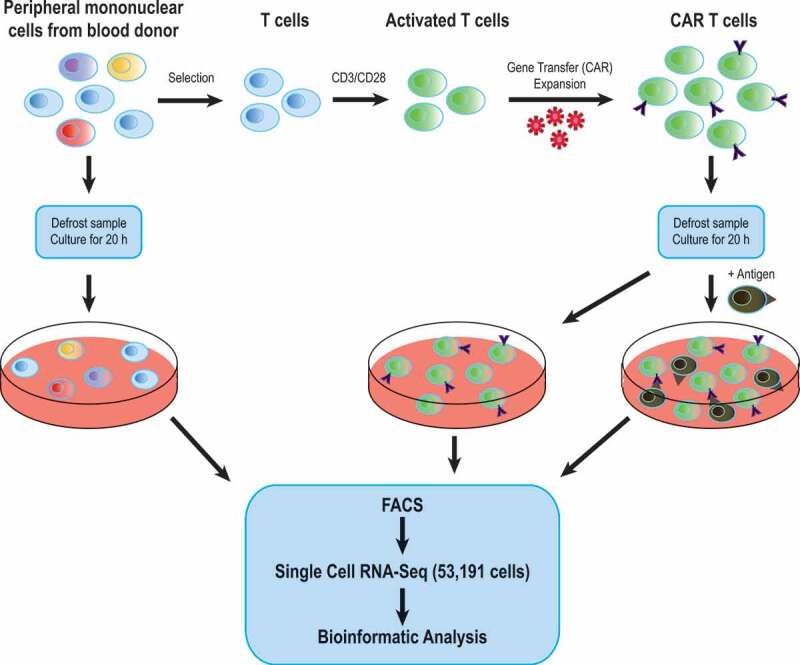
Experimental pipeline for single cell transcriptomic analysis of CAR T-cells. PBMCs from 3 different donors were obtained and T-cells were specifically activated using CD3/CD28 for 2 days prior to transduction of the chimeric antigen receptor (CAR). T-cells were then expanded and final product was frozen. For analysis, product samples were thawed, split in 2 and cultured overnight either in the presence or absence of the specific antigen. Aliquots of the original PBMC samples were thawed and cultured overnight. Samples were FACS sorted to remove dead cells followed by single cell RNA-Seq and bioinformatics analysis

To interrogate the molecular consequences of specific CAR activation, the CAR-T product from 3 healthy donors were generated and analyzed using a combined approach of traditional flow cytometry and scRNA-Seq. Each CAR product sample was split in half, with one half cultured in the presence of cells displaying the specific CAR antigen (Figure 1). This sampling strategy was designed to provide valuable information about (i) the similarity or otherwise of CAR products generated from different donors, (ii) a full molecular characterization of the CAR activation process, and (iii) to provide an opportunity to explore clinically relevant aspects such as the identification of possible subpopulations associated with exhaustion processes of activated CAR-T cells.

To obtain a better understanding, we analyzed the whole product containing a mixture of transduced and non-transduced cells. In this way, we could analyze the behavior of CAR-T cells within the context of non-transduced cells and obtained an internal reference for comparative analysis. We obtained the transcriptional profiles of 37,898 single cells corresponding to the CAR products of the three donors, of which 17,163 cells were from the product in the absence of CAR-specific stimulation and 20,735 cells from the product in the presence of CAR-specific antigen exposure (Table 1).

**Table 1.** Distribution of single cell transcriptomes analyzed in this work

	Unstimulated CAR-non-expressing			Unstimulated CAR-expressing			Stimulated CAR-non-expressing			Stimulated CAR-expressing			
	Donor 1	Donor 2	Donor 3	Donor 1	Donor 2	Donor 3	Donor 1	Donor 2	Donor 3	Donor 1	Donor 2	Donor 3	Total
Cluster 1	10	47	57	47	61	195	336	190	351	768	435	901	3398
Cluster 2	36	2	9	317	12	93	40	9	8	134	4	39	703
Cluster 3	397	162	61	201	90	25	404	101	37	70	25	4	1577
Cluster 4	134	1112	1793	32	173	561	203	1847	1997	28	133	286	8299
Cluster 5	1891	1139	1171	653	344	348	805	638	549	194	87	101	7920
Cluster 6	398	229	552	117	65	141	2636	970	1155	338	140	166	6907
Cluster 7	26	37	199	7	8	45	70	27	32	9	2	6	468
Cluster 8	19	12	41	10	6	18	24	13	20	7	0	4	174
Cluster 9	23	266	104	5	39	29	34	435	128	5	38	45	1151
Cluster 10	634	1291	986	266	201	207	760	1836	846	72	92	83	7274
Cluster 11	0	9	0	0	0	0	0	14	2	0	1	1	27
Total	3568	4306	4973	1655	999	1662	5312	6080	5125	1625	957	1636	37898
	12847			4316			16517			4218			

The mapping of the region upstream of the predicted polyadenylation site of the 3ʹLTR of the virus used to express the CAR allows the robust detection of CAR transcripts independently of the insertion point (Supplementary Figures 1A and 1B). CAR expression was detected on a total of 8,534 cells, of which 4,218 cells corresponded to antigen-exposed cells and 4,316 cells to unstimulated cells (Table 1). The CAR could be detected on an average of 22.5% of the cells, in line with previous reports,^2^ with expression levels similar in all samples (Supplementary Figure 1C). CAR-expressing cells also express RQR8 on the surface, which allows the detection of successfully infected cells by flow cytometry. Importantly, the percentage of CAR-expressing cells was very similar when measured by either scRNA-Seq or RQR8 detection by flow cytometry (Supplementary Figure 1D).

**Figure 2.**
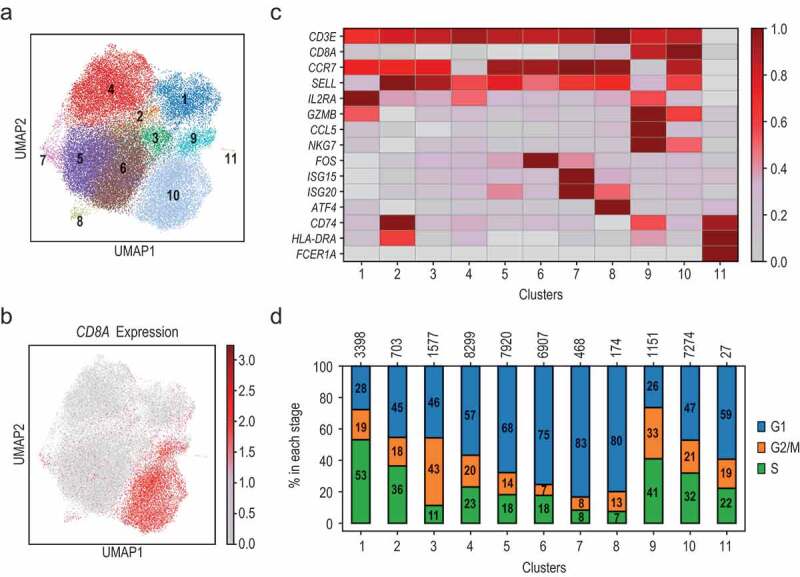
Single cell transcriptomics defines cellular composition of CAR product. (a) UMAP visualization of transcriptomic profiles of CAR product cells (contains CAR-expressing and -non-expressing cells before and after CAR-specific stimulation) and Leiden clustering. (b) The cells in the UMAP were colored according to the expression levels of *CD8A*. Color scheme is based on ln scale of normalized counts from 0 (gray) to the indicated maximum value in the scale (dark red). (c) Heatmap using selected genes to characterize each cluster. For each gene, mean expression in all cells within each cluster was calculated, scaled across the clusters and expressed relative to the maximum mean value. Color scheme goes from 0 (gray) to 1 (dark red). (d) Stacked bar chart showing the proportion of cells predicted in each cell cycle stage for each cluster. The total number of cells in each cluster is indicated at the top

Bioinformatic identification of cell clusters was used to identify cellular subtypes and/or molecular states present in the products, as defined by single-cell gene expression analysis. The clustering results were visualized using UMAP and the cells were colored retrospectively according to their allocated cluster. This analysis highlighted the presence of eleven clusters (Figure 2(a), Table 1). We next investigated the nature of the defined clusters using the expression of known T-cell subpopulation marker genes such as *CD8A, CD4, CCR7*, and *SELL* (CD62L)^8^ (Figure 2(b,c), Supplementary Figures 2A and 2B) and inferred the probability for each cell to stay in a particular cell cycle stage (Figure 2(d) and Supplementary Figure 2E) using a list of cell cycle genes previously defined.^9^ *CD8A* expression was strongly detected in clusters 9 and 10 whereas *CD4* expression was detected in the rest of the clusters. Clusters 1, 7, and 8 contained a mixture of *CD4* and *CD8*-expressing cells. Thus, we could define cells that expressed: CD4+CCR7^high^SELL^high^ (cluster 5); CD4+CCR7^high^SELL^mid^ (cluster 6); CD4+CCR7^low^SELL^mid^ (cluster 4); CD4+CCR7^high^SELL^high^ (clusters 2 and 3); CD8+CCR7^low^SELL^low^ (cluster 9); CD8+CCR7^high^SELL^high^ (cluster 10). Cells in cluster 1 presented high levels of *CCR7*, low levels of *SELL*, and higher levels of genes related to T-cell activation such as *IL2RA* (CD25) (Figure 2(c)).

All cells in the dataset expressed *CD3E* except a small number of cells that belonged to cluster 11 and a very small number of cells located within cluster 9 (Supplementary Figure 2C). Cells within cluster 11 expressed high levels of MHC class II (*HLA-DR, HLA-DP*, and *HLA-DM*) as well as *FCER1A* suggesting that they have a dendritic phenotype. The small number of cells in cluster 9 that did not express *CD3E* had a high expression of *GNLY, NCAM1*, and *NKG7* suggesting that they present a phenotype similar to NK cells (Supplementary Figure 2D).

### CAR products from different donors show a similar cellular composition

To assess the variability between CAR-T products from different donors in terms of cell types and distribution of cellular states, we compared the distribution of the three different donors within the previously defined clusters of CAR products.

**Figure 3.**
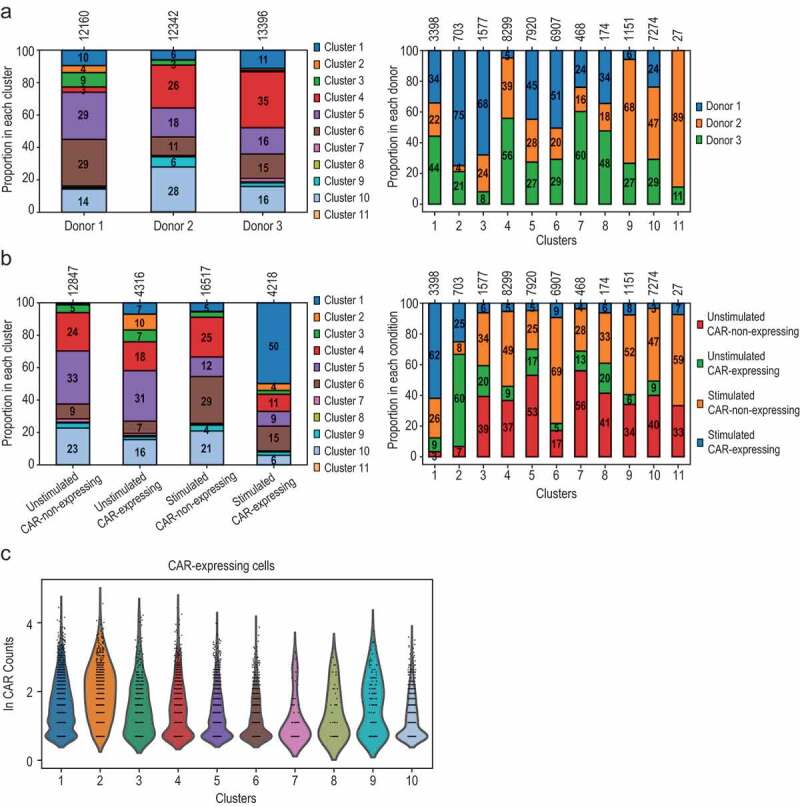
Cluster composition is independent of donor and condition. (a, b) All donors and all conditions contributed to all clusters. Of note, the small cluster 11 (only contained 27 cells) was the only exception. Distribution of cells per donor and cluster (a) and per condition and cluster (b) is depicted. The total number of cells in each group is shown at the top. (c) Violin plots showing expression levels (ln scale of raw counts on y axis) of CAR across CAR-expressing cells from the clusters. Cluster 11 was excluded since it only contained 2 CAR-expressing cells

Importantly, all clusters (except cluster 11 that only comprised 27 cells) contained cells from all three donors (Figure 3(a) and Supplementary Figure 3A), indicating that the scRNA-Seq data produced with our experimental and processing pipeline can be readily compared across different donors. The contribution of each donor to each cluster was variable, suggesting that the different subtypes/states can be present in different proportions in each donor. In particular, donor 1 was less represented in cluster 4 but contributed proportionally more to clusters 5, 6 and especially to cluster 2, where it constituted 75% of the cells within this cluster (Figure 3(a)).

### Early activation promotes effector-like transcriptional signatures

To gain a better understanding of the potential impact of the CAR T-cell production process (early activation treatment and transduction) on the cells, we compared the CAR product single-cell transcriptomes to those of the T-cells in the original PBMC starting material. To this end, we characterized 10,845 leukapheresis T-cells from the very same three healthy donors using scRNA-Seq analysis and identified 3,693 T-cells from donor 1, 3,763 T-cells from donor 2, and 3,389 T-cells from donor 3 (Extended Results). The UMAP visualization containing PBMCs and product T-cells separated cells according to their source (Supplementary Figures 4A and 4B) independently of the expression pattern of markers such as *CD8A, CCR7*, and *SELL* (Supplementary Figure 4C). We then integrated both datasets (Supplementary Figures 4D, 4E and 4F) and assigned leukapheresis T-cells to the closest predefined product T-cell clusters (Supplementary Figure 4G). Next, we compared unstimulated CAR-non-expressing cells and leukapheresis T-cells within each associated cluster (Supplementary Table 1). Of note, we could not identify a counterpart to cluster 1 in the leukapheresis sample, as can be observed from the pattern of expression of markers such as *IL2RA* (Supplementary Figure 4F), nor was there a clear separation between clusters 5 and 6 (Supplementary Figure 4E). We therefore excluded cells assigned to cluster 1 from this comparative analysis and we considered cells assigned to clusters 5 and 6 as one single group.

The integration of the 200 most upregulated genes from each of the different comparisons defined 5 groups of genes (Supplementary Figure 4H and Supplementary Table 1). One group of genes was upregulated in all clusters suggesting the presence of a common differentially expressed signature between the leukapheresis T-cells and product T-cells. This group contained genes that are upregulated in effector and effector memory cells when compared to naïve cells (“GSE11057_NAIVE_VS_MEMORY_CD4_TCELL_DN”, *p*-adjusted value 5.63E-11; “GSE9650_NAIVE_VS_EFF_CD8_TCELL_DN”, *p*-adjusted value 5.63E-11). Although we could define groups of genes upregulated in the specific clusters, the genes we identified were generally related to an acquisition of effector and effector memory states.

### CAR-expressing and CAR-non-expressing cells are similar in the absence of CAR-specific stimulation

Since we had determined that CAR-expressing cells can be robustly detected and we had generated parallel scRNA-Seq datasets for CAR products with and without CAR-specific antigen exposure, we then analyzed the distribution of CAR-expressing cells in the previously defined clusters to investigate whether CAR-expressing cells are enriched in certain subpopulations, and whether expression of the CAR influences transcriptional profiles even in the absence of antigen stimulation. Unstimulated CAR-expressing cells had a very similar distribution to the unstimulated CAR-non-expressing cells (Figure 3(b) and Supplementary Figures 3B and 3C). These results indicate that all major cell subtypes are equally susceptible to CAR virus transduction.

We then compared the unstimulated CAR-expressing and CAR-non-expressing cells within each of the major clusters to investigate if there were transcriptional differences between them. We could not detect a shared signature across the different comparisons and only few genes were differentially expressed for each of the clusters (Supplementary Table 2).

Our results suggest that CAR-expressing cells behave similarly to untransduced cells in resting conditions.

### Few CAR-expressing cells show transcriptional response without specific antigen stimulation

The proportion of CAR-expressing cells in the absence of specific stimulus was higher in clusters 1 and 2 when compared to non-expressing CAR cells (Figure 3(b) and Supplementary Figure 3C). Cluster 2 contained 422 unstimulated CAR-expressing cells (9.8% of 4316 unstimulated CAR-expressing cells) that presented a central memory-like phenotype (CCR7+ SELL^high^) although these cells were present mainly in only one of the donors (see Figure 3(a)). Cluster 1 (which was consisted mostly of CAR-expressing antigen-exposed cells) contained 303 unstimulated CAR-expressing cells (7% of all unstimulated CAR-expressing cells). These cells presented an activation signature that was independent of the CAR-expression levels (Figure 3(c) and Supplementary Figure 3D). Of note, unstimulated CAR-non-expressing cells could also be detected within clusters 1 and 2, although in much smaller proportions, 0.9% and 0.4%, respectively (114 and 47 cells out of 12,847 cells, respectively).

Our approach using single-cell transcriptomics has allowed us to reveal that approximately 7% of CAR-expressing cells are activated already prior to encountering the specific antigen.

### Antigen exposure results in homogeneous activation of CAR-expressing cells

Our sampling strategy allows us to compare the effect of exposure to the CAR-specific antigen in CAR-expressing T-cells, using non-expressing cells as a reference. Upon CAR-specific antigen stimulation, there is a relative increase of CAR-non-expressing cells in clusters 1 and 6 paralleled by a relative decrease of cells in cluster 5 when compared to the distribution in the absence of the stimulus (Figure 3(b) and Supplementary Figure 3C).

Cells in cluster 6 present lower levels of *SELL* and higher expression of genes that code for granzymes (*GZMA, GZMB*, and *GZMH*), cytokine genes (such as *IL3, IL4, IL5, IL8, IL13*, and *CSF2*), activating transcriptional factors (such as *FOS*, a member of the AP-1 complex) and receptors (such as *CCR1* and *CXCR6*) when compared to cluster 5 (Supplementary Table 3). These results suggest that a proportion of CAR-non-expressing cells acquired a transcriptional state resembling activation upon CAR-specific antigen stimulation, although at lower levels than cells in cluster 1. Since CAR-non-expressing cells cannot respond to the CAR-specific antigen, this would suggest that there is cell-to-cell signaling between the CAR-expressing cells and a proportion of CAR-non-expressing cells upon stimulation.

The distribution of CAR-expressing cells changed drastically upon stimulation. These cells were substantially enriched in cluster 1, where they constituted 62% of the cells, and dramatically reduced in all the remaining clusters, with the exception of a moderate increase in cluster 6 (in a similar trend to CAR-non-expressing cells) (Figure 3(b) and Supplementary Figure 3C). Our results suggest that a proportion of CAR-expressing cells acquire a similar transcriptional state upon antigen-specific stimulation and these cells simultaneously transition to cluster 1 from most of the other clusters.

**Figure 4.**
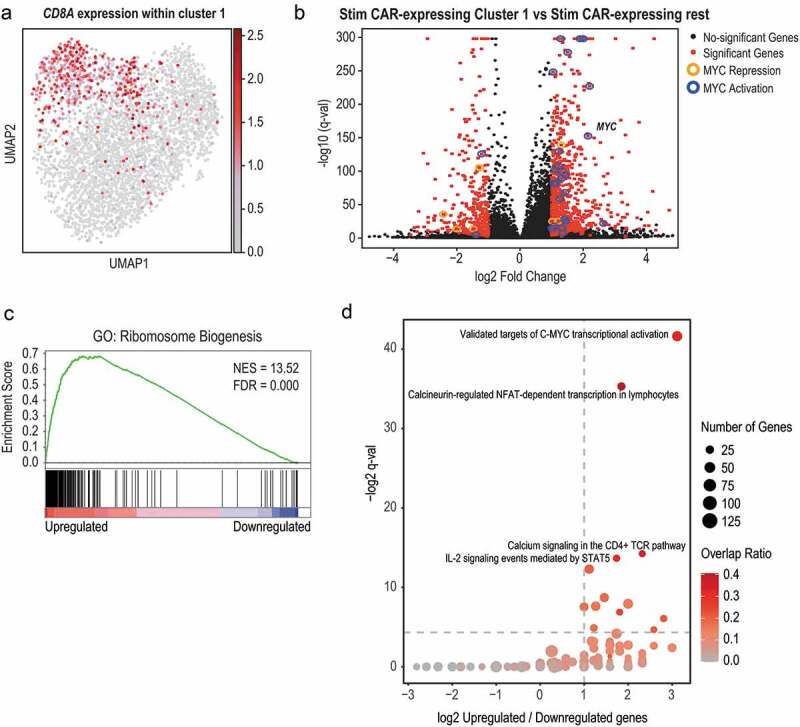
Antigen exposure results in homogeneous activation of CAR-expressing cells. (a) UMAP visualization of cells within cluster 1, which contains activated cells upon CAR-specific antigen stimulation. Cells were colored according to the expression levels of *CD8A*. Color scheme is based on ln scale of normalized counts from 0 (gray) to the indicated maximum value in the scale (dark red). (b) Volcano plot showing differentially expressed genes in antigen-exposed CAR-expressing cells contained in the activated cluster 1 compared with the antigen-exposed CAR-expressing cells contained in the rest of clusters. Fold change is presented in the x-axis (expressed as log 2) and the q-value of the analysis (expressed as – log10 of q-value) in the y-axis. Genes with log2 fold change > |1| and *p*-adjusted value < 0.05 and with log2 mean expression > −5 are depicted in red. Genes that overlapped with GO terms “Validated targets of C-MYC transcriptional activation” and “Validated targets of C-MYC transcriptional repression” are highlighted in blue and orange, respectively. (c, d) Gene set enrichments analysis (GSEA) of differentially expressed genes showed in B) with GO term “ribosome biogenesis” (c) and all GO terms contained in the PID database (d). In C, genes were ranked according to their *p*-adjusted value with most upregulated genes to the left and most downregulated genes to the right. Normalized Enrichment Score (NES) and Fold Discovery Rate (FDR) are shown. In (d), the ratio of overlapping upregulated and downregulated genes within each GO term is presented in the x-axis (expressed as log 2) and the q-value of the analysis (expressed as – log2 of q-value) in the y-axis. Thresholds indicating 2-fold upregulated genes over downregulated genes and minimum significance (FDR<0.05) are denoted. The size of the dot represents the number of genes included in the term. The dots are colored according to the ratio of upregulated genes that overlap relative to the total number of genes included in the term

Cluster 1 contained a small proportion of unstimulated cells and 26% of cells in this cluster corresponded to stimulated CAR-non-expressing cells (Figure 3(b)). We therefore investigated if the cells within cluster 1 had a homogenous transcriptional profile. To this end, we obtained an UMAP visualization of the cells within cluster 1 and could observe a separation between *CD8*- and *CD4*-expressing cells that could be confirmed by the appearance of subclusters within cluster 1 (Figure 4(a) and Supplementary Figures 5A and 5B). Of note, we could not see a separation between CAR-expressing and CAR-non-expressing cells or in relation to CAR-expression levels (Supplementary Figures 5D and 5E).

We also compared the expression patterns of *CD8*-expressing cells with the rest of the cells within cluster 1 and we found very few differences (Supplementary Figure 5C and Supplementary Table 4). These results suggest that there is one single program for T cell activation triggered by antigen stimulation and the presence of CAR, although a small proportion of cells can activate this program even in the absence of the antigen. Taken together, our analysis shows that nearly 50% of CAR-expressing cells respond to CAR-specific stimulation in a consistent fashion, thus implying the existence of a molecular signature that should be characteristic of the CAR response to specific antigen.

### Some CAR-expressing cells show no transcriptional response to specific antigen stimulation

Our previous results revealed a specific transcriptional response in a subset of CAR-expressing cells upon exposure to specific antigen stimulation. However, a big proportion of CAR-expressing cells from the stimulated condition were assigned to other clusters than cluster 1 where they were intermixed with non-CAR expressing cells in both clustering and UMAP analysis, thus indicating that CAR-expressing cells within those clusters do not respond to antigen exposure. We then investigated whether these cells did not present a transcriptional response to the antigen stimulation or whether they showed evidence of a short-term response to the stimulus. Since we had cultured the product in parallel in the presence and absence of the CAR-specific antigen, we next compared the unstimulated and stimulated CAR-expressing cells within clusters other than cluster 1. We could not find major differences between these cells (Supplementary Table 5). Our results suggest that cells in the product exposed to antigen that fall outside of cluster 1 remain transcriptionally very similar to cells that have not been exposed to the antigen.

### The molecular signature of CAR activation includes upregulation of the MYC program

To dissect the molecular program of CAR activation, we focussed our bioinformatic analysis on the 4,218 stimulated CAR-expressing cells. We compared the transcriptional profiles of antigen-exposed cells present in cluster 1, which contained the activated cells, with antigen-exposed cells present in all the other clusters. Differential gene expression analysis showed that 899 genes were upregulated and 364 genes downregulated in antigen-exposed cells within cluster 1 when compared with the other clusters (Figure 4(b) and Supplementary Table 6). The upregulated genes included receptors (such as *CCR4*), cytokines (such as *IL2, IL3, IL4, IL5, IL8, IL10, IL13*, and *CSF2*) and granzyme B (*GZMB*). Analysis of the upregulated genes showed an enrichment in genes corresponding to pathways related to lymphocyte activation (Figure 4(c,d)). Enriched pathways included “ribosome biogenesis” (*p*-adjusted value 5.29E-76), genes related to the “calcineurin-regulated NFAT-dependent transcription in lymphocytes” (*p*-adjusted value 2.35E-11) (which contains genes such as *BATF3, CSF2, IRF4, CDK4, IL2RA, IL3, IL4*, and *IL5*) together with “Calcium signaling in the CD4+ TCR pathway” (*p*-adjusted value 5.19E-05) and “IL-2 signaling mediated by STAT5” (*p*-adjusted value 7.72E-05). *MYC* was also found to have a prominent role in the CAR activation (*p*-adjusted value 2.96E-13). Not only is *MYC* itself upregulated but additionally validated target genes of MYC were also upregulated (including genes such as *FOSL1, CDK4, KAT2A, PMAIP1, CDC25A, NME1, NPM1, TFRC*, and *BMI1*).

The downregulated genes contained expected genes such as *SELL*^10^ and genes that code for MHC class I, such as *B2M*. Of note, genes such as *HLA-A, HLA-B, HLA-C, HLA-E*, and *HLA-F* had an FDR <0.05 and a fold-change close to −2. In summary, our results show that CAR-expressing cells activated upon antigen exposure activate similar pathways to the ones triggered by the T-cell receptor.

### Only a small proportion of stimulated CAR-expressing cells exhibit exhaustion features

**Figure 5.**
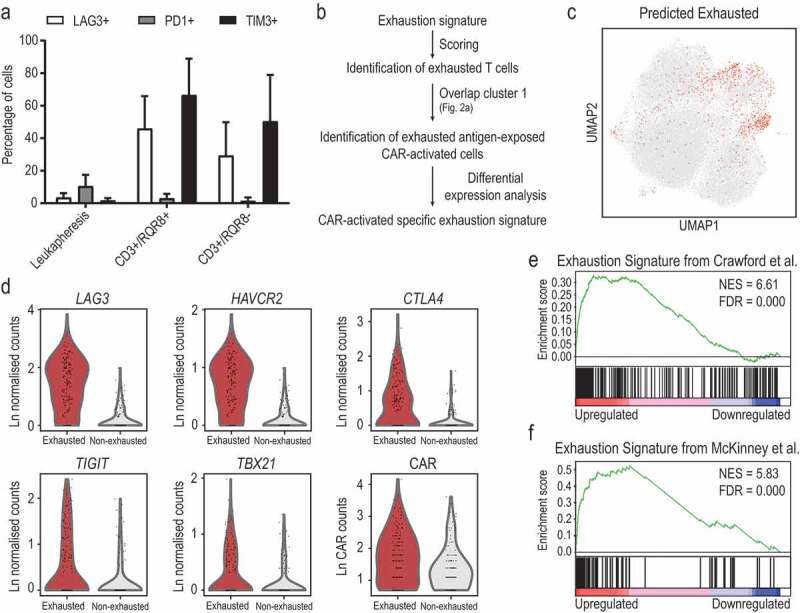
Exhaustion is only a minor consequence of CAR activation. (a) Detection of typical exhaustion markers by flow cytometry in leukapheresis, CAR-expressing and non-expressing product cells. (b) Strategy followed to identify putative exhausted cells and their transcriptional signature. (c) Distribution of putative exhausted cells in the UMAP visualization showing all product cells in the study. (d) Expression of genes linked to in the 153 most putative exhausted cells compared to 153 less likely exhausted cells based on the exhaustion scores (cluster 1 of Figure 2(a)). (e, f) Gene set enrichment analysis of differentially expressed genes in the 153 most putative exhausted cells compared to 153 less likely exhausted cells based on the exhaustion scores. Genes are pre-ranked according to their significance in the analysis, most upregulated to the left and most downregulated to the right. Normalized Enrichment Score (NES) and Fold Discovery Rate (FDR) are shown

The exhaustion of T-cells commonly occurs upon long exposure to the antigen in the absence of adequate costimulatory signals. It is an important factor to take into consideration when producing CAR T-cells since there are reports of relapse after an initial phase of clearing of the disease followed by the eventual exhaustion of CAR T-cells.^11^ Traditionally, surface markers including PD-1, TIM-3 and LAG-3 have been used to identify exhausted T-cells by flow cytometry. We measured the expression of those surface markers in the lymphocytic compartment of our leukapheresis and final product by flow cytometry (Figure 5(a) and Supplementary Figure 6A). Very few cells expressed the inhibitory receptor PD-1 in the final product and only a slightly higher proportion of cells expressed it in the original leukapheresis samples. TIM-3 and LAG-3 were detected in a higher proportion of cells of the final product than the leukapheresis with no differences between CAR-expressing and non-expressing cells.

From a transcriptomic point of view, exhaustion is characterized by the upregulation of inhibitory receptors and downregulation of stimulatory signals. A molecular signature for exhaustion was defined for mouse upon chronic viral infection,^12^ and has also subsequently proved to be informative in patients with autoimmune diseases^13^ as well as in the response of CML patients to treatment with anti-CD19 autologous CAR-T cells.^14^ We used this molecular signature to evaluate the exhaustion state of cells in our dataset by scoring all single-cell transcriptomes from the final products in our study against these genes (Figure 5(b), Supplementary Table 7 and methods). This comprehensive transcriptome-based approach identified 752 cells with a strong exhaustion gene signature (Figure 5(c) and Supplementary Figure 6B). These cells showed a good overlap with the expression of typical markers used to evaluate exhaustion by flow cytometry, particularly *LAG-3* (Supplementary Figure 6D), and the ones located within cluster 1 were mainly concentrated within one of the previously defined subclusters for cluster 1 (Supplementary Figures 5B and 6C). Only 7% (153 cells out of 2,104) of the responding antigen-exposed CAR-expressing cells (cluster 1 in Figure 2(a)) presented an exhaustion signature. Importantly, these 153 cells presented higher expression levels of the CAR when compared with non-exhausted-stimulated CAR-expressing cells within the same cluster (Figure 5(d)). Another noteworthy observation is that the exhaustion signature cells were distributed unequally between the donors, with most coming from donor 3, fewer from donor 1 and even fewer from donor 2 (Supplementary Figure 6E). The frequency of these cells may therefore represent a key feature that distinguishes CAR T-cell products from different donors.

We next compared the 153 CAR-expressing cells with strong exhaustion signature from cluster 1 to the 153 CAR-expressing and antigen-responding cells with the lowest exhaustion score from the same cluster (Supplementary Table 8). The cells with high exhaustion signature showed upregulation of typical co-inhibitory receptors (like *LAG3, HAVCR2*/*TIM3, CTLA4*, and *TIGIT*) (Figure 5(d)). Moreover, differential gene expression analysis demonstrated overexpression of gene sets previously shown to be expressed by exhausted CD4 and CD8 murine T-cells^15^ (Figure 5(e)). The differentially upregulated genes included typical exhaustion transcription factors (like *T-BET*/*TBX21*^15^) (Figure 5(d)), *IFNG*, chemokine genes (such as *CCL1, CCL5, CCL3*/*MIP1-alpha*, and *CCL4*/*MIP1-beta*), genes specifically identified in exhausted CAR-T cells (such as *ENTPD1*^16^), as well as genes downregulated following a treatment to prevent exhaustion of human CD8 T-cells^13^ (Figure 5(f)).

In summary, our data not only indicate that a small proportion of CAR-expressing cells exhibit features similar to T-cell exhaustion following antigen-specific CAR stimulation, but also that scRNA-Seq represents a powerful analytical technique to (i) quantify their proportion in CAR products, and (ii) identify previously unrecognized genes affected by immune cell exhaustion, with direct implications for both correlation to patient outcomes as well as optimization strategies for product development.

## Discussion

Here we provide a comprehensive single-cell transcriptomic analysis of key sequential stages during CAR T-cell generation. Traditionally, the characterization of immune cell populations has relied on flow cytometry, which resulted in a detailed vocabulary to describe primary immune cell populations. However, the link between surface marker expression and cellular function is often lost during *in vitro* culture, thus making a nomenclature based on surface markers potentially unreliable. Moreover, the need to pre-select markers from a list of ready-made antibodies prohibits a data-driven approach, which would collect unbiased information at full genome-scale, and then “learn” the subpopulation structure purely from the data itself. Here we used flow cytometry analysis to define basic parameters of CAR T-cell populations, and then performed comprehensive scRNA-Seq analysis, which allowed us to show that overall cellular composition is reproducible between donors. We furthermore show that all major cell populations are transduced with the CAR virus, yet only a subset of CAR-expressing cells responds to antigen-mediated activation. Moreover, we devised a new bioinformatic pipeline to define CAR activated cells displaying an exhaustion signature, thus providing a new means to assess this clinically relevant subpopulation in CAR T-cell products.

Our results show that the CAR product is heterogeneous and that cell cycle and predicted memory status are important factors to define this heterogeneity, in line with previous reports.^8,17^ Interestingly, we find that this heterogeneity is present in all donors although at different levels. We also corroborated that all major subpopulations present after T-cell culture were susceptible to CAR transduction as reported before,^18^ and moreover found that approximately 7% of CAR-transduced cells already presented an activation signature before antigen-specific stimulation. Importantly, high levels of CAR expression are unlikely to cause this activation in the absence of antigen since CAR expression in pre-activated cells is not higher than in cells without this gene signature. The reports of CAR T-cell activation are contradictory; some report homogenous response^17^ while others report diverse signatures.^19^ In our study, stratifying cell populations in a data-driven way based on their full transcriptomes allowed us to demonstrate that, following antigen activation, all major subpopulations within the CAR product can respond to antigen-specific stimulation in a similar way, thus revealing a uniform activation signature, which included a strong *MYC* gene signature together with the upregulation of genes related to calcium activation pathways. Both pathways are usually associated with an increase of glycolysis following T-cell stimulation.^20^ We also detected concomitant downregulation of *CD3D, CD3E* and *CD3G* (components of the T cell receptor) (Supplementary Table 6) which is well-established upon T-cell response^21^ as well as upregulation of genes involved in ribosomal RNA synthesis and processing.^22^ We also detected a strong upregulation of *CSF2, IL2RA*(CD25) and cytokines as previously described in cytolytic CAR T-cells.^19^

Of note, we did not detect expression of CD8 in the majority of CAR cells responding to antigen stimulation. The existence of CD4 CAR T cells with lytic activity has been described before.^18,19^ These cells present a very similar behavior and transcriptional profile to CD8 CAR T cells with lytic activity,^19^ although they have been suggested to have weaker activity than CD8 cells.^18^ Interestingly, previously reported CD4 CTL may be related to the CD4-expressing CAR T-cells in our samples. CD4 CTL cells express high levels of *GZMB* and *IFN*G.^23^ Accordingly, we detected increased expression of *BATF, BATF3*, and *IRF4* which are involved in the differentiation of effector T-cells^24,25^ and collaborate to promote genes such as *T-BET* (*TBX21*), which was also upregulated, and has been shown to promote the production of CD4 CTL cells.^23^ These comparisons not only provide new hypotheses for future investigations, but also demonstrate that the cellular complexity of CAR products may be higher than previously anticipated.

The conventional identification of memory cells using flow cytometry relies on detecting different isoforms of the gene *PTPRC* (that codes for CD45), but this portion of the mRNA is covered with very low efficiency when using droplet scRNA-Seq technology that captures the 3ʹ end of the mRNA. The expression of *CCR7* and *SELL* has been used in the past to identify different cell types in datasets generated using this technology.^8^ The bioinformatic pipeline devised here detects *CCR7* expression at the same proportion as identified by flow cytometry thus providing independent validation. The memory status of T-cells before CAR production is thought to represent a key parameter when trying to predict the downstream performance of the therapeutic product. Detailed attention has also been paid to the ratios of CD4:CD8 cells and the proportion of different subpopulations within the product.^18,26^

In our study, we combined the expression of *CCR7* and *SELL* with unsupervised clustering to group cells with similar transcriptional profiles. This analysis demonstrated that memory status is a strong parameter contributing to cell grouping, as is the CD8 or CD4 nature of the cells. Importantly, our data-driven approach allowed us to account for heterogeneity within and between donors, and thus derive broadly relevant cell classifications that would not have been possible to obtain using conventional approaches.

The exhaustion status of T-cells has previously been linked with the *in vivo* response of CAR T cells.^14^ Robust ways of identifying cells with exhaustion status within CAR products therefore represents an important goal with broad clinical relevance. We devised a bioinformatic pipeline that allows us to score single-cell transcriptomes based on the activity of a previously defined gene signature in chronically stimulated murine T-cells.^12^ This analysis identified a subset of cells, which showed specific upregulation of genes that matched previously described exhaustion signatures.^13,15^ Only few cells showed an exhaustion signature, corroborating previous reports of low incidence of exhaustion following acute stimulation in CAR-activated cells.^17,19^ Single-cell molecular profiling therefore emerges as a potentially powerful analytical technique, that can be used to define coarse grain population structure, donor-specific differences in subpopulation abundance, and also functional parameters such as the proportion of cells displaying an exhaustion signature. Application to extensive clinical studies would provide unprecedented new opportunities to correlate CAR product features with clinical outcomes, and thus guide patient management as well as the design of new and improved CAR production protocols.

## Materials and methods

### Donor samples

PBMCs were derived from healthy donors leukapheresis. Peripheral blood leukapaheresis were obtained from the NHS as part of a research study (IRAS ID 185945) or purchased as LeukoPaks from AllCells.

### Manufacturing of CAR T cells product

Genetically modified T cells were generated using the CliniMacs Prodigy (Miltenyi) following manufacturer’s instructions. Transduction was performed using an APRIL-CAR retroviral vector as previously described.^7^ The sequence of the CAR corresponds to SEQ ID No. 17 found within the patent WO 2015/052538. Briefly, T-cells are enriched from leukapheresis blood samples by stimulating their proliferation through 2 days of specific T-cell stimulation (CD3/CD28). Following removal of the stimulus, cells are infected with a retrovirus containing the CAR and cultured for 5 days in the presence of cytokines to promote T cell expansion, after which the culture is entirely made up of T cells. The final product is then stored in liquid nitrogen as well as the bulk of the original leukapheresis sample (Figure 1). CAR transduction efficiency was assessed in the final product by flow cytometry using RQR8 staining (Qbend10 antibody; R&D).

### Target cells

MM.1S cells were obtained from ATCC (CRL-2974) and cultured in RPMI (Lonza) supplemented with 10% FBS (Gibco) and 1% Glutamax (Gibco).

### Sampling for analysis

Leukapheresis samples were thawed, washed and seeded at a concentration of 2 × 10^6^ cells/ml in TexMacs (Miltenyi) supplemented with human AB serum (Seralab) and IL-7 and IL-15 (Miltenyi) in 24 well plates.

Similarly, product samples were thawed, washed, and resuspended at a concentration of 1 × 10^5^ CAR^+^ cells/ml in 0.5 ml of culture media and cultured for 24 hours in 48 well plates. For the antigen-exposure experiments, product samples were co-cultured in the presence of MM1.S cells in a 1:1 effector (CAR^+^ cells)/target (MM1.S) ratio.

For single-cell RNA-Seq analysis, following 20 hours of incubation, wells from the same conditions were pooled and all samples were FACS sorted, selecting live cells by using DAPI staining. Cells were washed and counted before entering the droplet-based scRNA-Seq workflow.

### Flow cytometry

Cellular composition of leukapheresis samples was determined by flow cytometry using the following antibodies (20 min at 4°C): CD3 (BD), CD19 (BioLegend), and CD56 (BioLegend).

Expression of exhaustion markers was determined by flow cytometry using the following antibodies (20 min at 4°C): CD3 (BD), RQR8 (Qbend10 antibody; R&D), CD8 (BioLegend), LAG3 (Enzo Life Sciences), PD1 (BioLegend), and TIM3 (BioLegend).

All samples were counterstained with 7AAD (BioLegend) to exclude dead cells.

BD Celesta was used for cell acquisition, and data were analyzed using FlowJo V10 (Treestar). Representative plots showing the gating strategy are shown in Supplementary Figure 6A.

### Droplet-based scRNA-sequencing

Samples were processed following manufacturer’s recommendations for Chromium Single Cell 3′ Library & Gel Bead Kit v2 (10X Genomics). 17,500 cells were loaded for each sample and 1 sample was loaded per condition. Samples were sequenced in Illumina HiSeq4000 sequencer machine. We obtained an average of ~286 million reads per sample. Per cell, we obtained an average of ~46,000 reads; 6,194 median UMIs and 1,851 median genes detected.

### Pre-processing of scRNA-seq data

The alignment was done using *Cellranger* (version 2.0.0). The expected number of cells was set to 10,000. In total, 17,089 cells were detected for leukapheresis samples and 40,523 cells for product T cells. The downstream analysis was done using *Scanpy*^27^ (version 1.5.0). For the leukapheresis samples, 382 doublets were estimated and removed using *Scrublet* package^28^ (version 0.2.1) in *Python*. Due to the homogeneity of the product samples, this method is not adequate for the estimation of doublets in these samples. Further quality control was performed based on 3 parameters: 1) at least 400 or 1,000 genes detected per cell for leukapheresis or product samples, respectively; 2) less than 8% of UMI counts associated to mitochondrial genes; 3) more than 10,000 or 30,000 of total UMI counts per cell for leukapheresis or product samples, respectively. After QC, 15,293 cells were remained for leukapheresis samples and 37,898 cells were remained for product T cells. In addition, only genes that have more than 1 UMI count were maintained in further analysis. Cells were then normalized to 10,000 UMIs per cell and logarithmically transformed. Highly variable genes (HVGs) were selected using “*highly_variable_genes”* method with “flavor *= ‘Seurat’, min_mean =0.02, max_mean =3, min_disp =.5”* with *“batch_key”* included so that the HVGs were selected within each batch separately and merged. Read depth, number of genes, number of mitochondrial counts and cell cycle effects were removed using the “*regress_out*” function in *Scanpy*. The number of cell barcodes retained for each sample/condition after processing can be found in Table 1.

As part of our scRNA-Seq processing pipeline, we mapped sequence reads in parallel to both the human reference genome and a customized reference genome that included the sequence encompassing the 1.3 kb just upstream of the predicted polyadenylation site of the 3ʹLTR of the virus used to express the CAR. This strategy allows the detection of CAR transcripts since the polyadenylation site located in the 3ʹLTR of the virus is used independently of the insertion point.

### Data integration

In order to directly compare between the leukapheresis and the product T cells, we used *BBKNN*^29^ (version 1.3.9) to calculate the batch-balanced neighbors between them with “*neighbors_within_batch*” set to “7”. Leukapheresis T cells were assigned to the most frequent product cluster of the closest 7 product neighbor cells.

### Visualization and clustering

UMAPs were obtained from 50 PCA components using *Scanpy. Louvain* clustering was used to obtain clusters in leukapheresis samples and *Leiden* clustering was used to obtain clusters in product T cells. Modularity scores were calculated from resolutions 0.1 to 2 and the final number of clusters were selected based on the trade-off of the modularity score and the biological complexity.

### Differential expression analysis

Differential expression analysis was done using “*rank_genes_groups”* function in *Scanpy* with method “*t-test”*. The *p*-values were adjusted using Benjamini-Hochberg method. Genes were considered differentially expressed only if complied with all the following criteria: i) FDR < 0.05; ii) log2 fold change > |1|; and 3) base mean expression > −5 (results in Supplementary Tables 1, 2, 4, 5 and 6) or > −7 (results in Supplementary Table 3) or > −3 (results in Supplementary Table 8).

To characterize each cluster, mean expression in all cells within each cluster was calculated for each selected gene and scaled across the clusters using “*matrixplot*” function with parameter “*standard_scale =’var’*” in *Scanpy*.

For the comparison between leukapheresis and product T cells, the 200 most upregulated genes from each cluster comparison were obtained and the union list was extracted. The upregulated genes from each cluster were converted into binary vectors so that if a gene overlaps with the union of genes, then 1 was assigned, otherwise, 0. The Euclidean distances between genes and clusters were calculated using “*pdist*” function from *SciPy* package (version 1.4.1) in *Python*. Then the hierarchical clustering was performed with ‘*ward*’ method using the “*linkage*” function in *SciPy*. The heatmap was plotted using “*clustermap*” function in *Seaborn* (version 0.10.0).

### Prediction of exhaustion status

The list of 107 up-regulated genes that constitute the previously defined exhaustion signature^12^ (Supplementary Table 7) was obtained and intersected with the list of highly variable genes in our dataset. The exhaustion score was calculated using the “*score_genes*” function in *Scanpy* based on the 26 overlapping exhaustion genes (Supplementary Table 7). The cells with exhaustion score larger than 0.6 were considered as cells with high exhaustion potential.

### Gene set enrichment analysis

The gene set enrichment analysis of upregulated genes in cluster 1 (relates to Figure 4d) was performed using the hypergeometric test (“*phyper”* function in R). The over-represented genes are determined by:

$$P\left(X\geq x\right)=HGT\left(x,N,m,k\right)=\overset{min\left(n,B\right)}{\underset{i=x}{\sum }}\frac{\left(\begin{array}{c} m\\ i \end{array}\right)\left(\begin{array}{c} N-m\\ k-i \end{array}\right)}{\left(\begin{array}{c} N\\ k \end{array}\right)}$$

where

- N: the total number of expressed *Homo sapiens* genes

- m: the number of genes in individual reference database terms

- k: the number of upregulated/downregulated genes

- x: the number of upregulated/downregulated genes found in individual reference database terms

*P*-values were then corrected using the Benjamini–Hochberg (BH) method for multiple comparisons.

All gene sets included in the Pathway Interaction Database (PID) were used for comparison. The PID gene sets with adjusted *p*-value lower than 0.05 were selected as being significant. The upregulated/downregulated ratio for the selected pathways was calculated as:

$$\frac{upregulated\;genes\cap genes\;in\;the\;sig\;PID\;pathway}{downregulated\;genes\;\cap genes\;in\;the\;sig\;PID\;pathway}$$

The calculation was performed using the in-house single-cell analysis pipeline *smqpp*^30^ (version 0.1.1) in *Python*.

### Pre-ranked gene set enrichment analysis

All genes were ranked according to the scores calculated using the following:

$$\frac{1}{adjusted\;pvalue}*sign\left(log2FC\right)$$

where *adjusted p-value* and *log2FC* values were obtained from the differential expression analysis.

The resulting pre-ranked gene lists were used as reference sets for GSEA Pre-ranked analysis using GSEA software (version 4.0.3) with parameters *enrichment statistic =’classic’, Max size =500 and Min size =15*. The lists of genes sets used for the comparison were: GO:0042254 “Ribosome Biogenesis”; upregulated genes in exhausted T-cells (Supplementary Table 7), obtained from Crawdford *et al*.;^15^ downregulated genes upon CD2-co-stimulation (Supplementary Table 7), obtained from McKinney *et al*.^13^

## Supplementary Material

Supplemental Material

Supplemental Material

## Data Availability

The data corresponding to the single-cell RNA-Seq is deposited in the Gene Expression Omnibus database (https://www.ncbi.nlm.nih.gov/geo/) with access number GSE145809. The code used for the analysis has been deposited in GitHub (https://github.com/SharonWang/CARTcells_Notebooks/).
