# Supplementary material for: Single-cell transcriptome analysis of CAR T-cell products reveals subpopulations, stimulation, and exhaustion signatures
Source: Oncoimmunology. 2021 Jan 6;10(1):1866287. doi: 10.1080/2162402X.2020.1866287 (PMC7801130; doi:10.1080/2162402X.2020.1866287)
Supplement: Supplemental Material [file KONI_A_1866287_SM5890.zip › supplementary figures/Wang_Extended Results.docx]

**Single Cell Transcriptome Analysis of CAR T-Cell Products Reveals Subpopulations, Stimulation and Exhaustion Signatures**

Xiaonan Wang^1^, Carlotta Peticone^2^, Ekaterini Kotsopoulou^2^, Berthold Göttgens^1*^, Fernando J Calero-Nieto^1*^

1: Wellcome and MRC Cambridge Stem Cell Institute and University of Cambridge Department of Haematology, Jeffrey Cheah Biomedical Centre, Puddicombe Way, Cambridge CB2 0AW, UK

2: Autolus Ltd, London, UK

*: Correspondence should be addressed to BG (bg200@cam.ac.uk) or FJC-N (fjc28@cam.ac.uk)

Wellcome and MRC Cambridge Stem Cell Institute and University of Cambridge Department of Haematology, Jeffrey Cheah Biomedical Centre, Puddicombe Way, Cambridge CB2 0AW, UK

Tel.: +44-1223-336829 FAX: +44-1223-762670

E-mail: BG (bg200@cam.ac.uk) or FJC-N (fjc28@cam.ac.uk)

Short Title: scRNA-Seq Characterisation of CAR T-Cell Products

**Extended Results**

**scRNA-Seq defined subpopulations are consistent between different leukapheresis samples**

To get a better understanding of the possible effects of inter-individual variability of leukapheresis samples, we characterised cells from three healthy donors using scRNA-Seq analysis. We obtained 5,184 cells from donor 1, 5,136 cells from donor 2 and 4,973 cells from donor 3. The single cell transcriptomes from all three peripheral blood mononuclear cell (PBMC) samples were merged, and the resulting 15,293 single cell transcriptomes were used to perform clustering analysis to identify major populations. The resulting clusters were then visualised using UMAP with the cells retrospectively coloured according to their allocated cluster (Extended Results Figure 1A). To classify each cluster, we investigated the expression of markers traditionally used to identify PBMC populations by flow cytometry (Extended Results Figures 1B and 1C).Cells within cluster 1 expressed high levels of *CD79A* and *CD19*, suggesting that they contain mainly B-lymphocytes. Cluster 2 contained cells that expressed high levels of myeloid markers, such as *FTL* and *S100A8*. Cells within cluster 3 expressed high levels of *GNLY* and *NKG7* but only a few cells expressed *CD3A*.

We identified clusters 4, 5, 6 and 7 as the clusters that contained the T-cells as identified by the expression of *CD3A* and either *CD8A* or *CD4*. A total of 10,845 cells were contained in these clusters, of which 3,693 cells were from donor 1, 3,763 cells were from donor 2 and 3,389 cells were from donor 3.

To complete the analysis of PBMCs, we assessed cell type composition in the different donors separately (Extended Results Figure 1D). All cell types were present in all three donors and the proportions were very similar in all donors except for the cluster corresponding to monocytes (cluster 2), which was specifically expanded in donor 1 when compared to the other donors.

Taken together, we show that scRNA-Seq can be used to define the cellular composition of PBMCs, and that this approach is robust in terms of inter-individual variation, thus providing a baseline for subsequent analysis of CAR cell products using the same technology.

**Extended Results Figure Legends**

**Extended Results Figure 1.- Single cell transcriptomics defines cellular composition of starting material.** Transcriptomic profiles from 15,293 PBMCs from 3 different donors were obtained, visualised using UMAP and separated using Louvain clustering **(A)**. **(B)** and **(C)** Clusters were classified using a combination of expression of traditional flow cytometry markers. **(D)** Pie chart representing the proportion of cells assigned to each of the different clusters based on their transcriptomic profile in each of the 3 donors. Percentages and absolute numbers of cells in each cluster are denoted.
