# Supplementary material for: Single-cell transcriptome analysis of CAR T-cell products reveals subpopulations, stimulation, and exhaustion signatures
Source: Oncoimmunology. 2021 Jan 6;10(1):1866287. doi: 10.1080/2162402X.2020.1866287 (PMC7801130; doi:10.1080/2162402X.2020.1866287)
Supplement: Supplemental Material [file KONI_A_1866287_SM5890.zip › supplementary figures/Wang_Supplementary Figure Legends_Resubmission2.docx]

**Supplementary Figure 1.- Detection of CAR. (A)** Distribution of genes according to the number of normalised mean of detected counts. Positions corresponding to *CD3* (red), *CD4* (yellow), *CD8* (blue) and CAR (green) are shown as dashed lines. X axis is expressed in log2 values. **(B)** Distribution of genes according to the maximum number of normalised detected counts. Positions corresponding to *CD3* (red), *CD4* (yellow), *CD8* (blue) and CAR (green) are shown as dashed lines. X axis is expressed in log2 values. **(C)** Violin plots showing expression levels (ln scale of raw counts on y axis) of CAR across antigen-exposed and antigen-non-exposed samples from the 3 patients. The value for each individual cell is shown. **(D)** Bar charts representing the percentage of CAR-positive cells detected in the antigen-exposed and antigen-non-exposed samples from the 3 patients measured by scRNA-Seq (grey bars) or flow cytometry (black bars). N=1 biologically independent sample.

**Supplementary Figure 2.- Characterisation of clusters of CAR product samples.** UMAP visualisation of single cell RNA transcriptomes obtained from product samples from 3 donors as shown in Figure 2A. The cells were coloured according to the expression levels of denoted genes markers *CD4*, *CCR7* and *SELL* **(A)**, *CD3D* ***(C)*** and *NKG7, GNLY* and *NCAM1* **(D)**. Colour scheme is based on ln scale of normalised counts from 0 (grey) to the indicated maximum value in each scale (dark red). **(C)** Violin plots showing expression levels (ln scale of raw counts on y axis) of *CCR7* (top panel) and *SELL* (bottom panel) across cells from the different clusters. The value for each individual cell is shown. Indicative lines for comparison of expression between clusters are shown. (E) UMAP visualisation of cells coloured according to their predicted cell cycle stage.

**Supplementary Figure 3.- Characterisation of CAR product samples by donor and condition.** UMAP visualisation of single cell RNA transcriptomes obtained from product samples from 3 donors as shown in Figure 2A. The contribution of each of the 3 donors **(A)** and each of the four conditions **(B)** is shown. **(C)** Proportion of cells of each condition present in each of the defined clusters. P-values were calculated using Mann-Whitney U test. Significance is shown, * denotes p-value < 0.05. **(D)** Violin plots showing expression levels (ln scale of raw counts on y axis) of CAR across antigen-exposed (right panel) and antigen-non-exposed (left panel) cells in each of the defined clusters. The value for each individual cell is shown.

**Supplementary Figure 4.- Comparison of T-cells from leukapheresis and CAR-products. (A)** UMAP visualisation of single cell RNA transcriptomes of T-cells from leukapheresis and all cells from product samples (including the four conditions). **(B)** Cells from product samples in the UMAP in (A) were coloured according to the previously defined clusters (Figure 2A). **(C)** The cells in the UMAP in (A) were coloured according to the expression levels of denoted gene markers *CD8A*, *CCR7*, *SELL* and *IL2RA*. Colour scheme is based on ln scale of normalised counts from 0 (grey) to the indicated maximum value in each scale (dark red). **(D)** UMAP visualisation of single cell RNA transcriptomes of T-cells from leukapheresis and all cells from product samples (including the four conditions) following integration. **(E)** Cells from product samples in the UMAP in (D) were coloured according to the previously defined clusters (Figure 2A). **(F)** The cells in the UMAP in (D) were coloured according to the expression levels of denoted gene markers *CD8A*, *CCR7*, *SELL* and *IL2RA*. Colour scheme is based on ln scale of normalised counts from 0 (grey) to the indicated maximum value in each scale (dark red). **(G)** Cells from product samples in the UMAP in (D) were coloured if belonged to either clusters 1, 4, 5, 6, 9 or 10. Clusters 5 and 6 were merged for this analysis (left panel). Leukapheresis T cells in the UMAP in (D) were coloured according to their associated cluster (right panel). **(H)** The union of the 200 most upregulated genes when comparing unstimulated CAR-non-expressing cells and leukapheresis T-cells within each associated cluster was used to perform unsupervised hierarchical clustering. The heatmap shows genes in the rows and clusters in the columns. Red colouring denotes presence of the indicated gene in the 200 most upregulated genes within the cluster. Five gene clusters were determined according to the expression pattern. These clusters are indicated on the left in blue, orange, green, red and blue.

**Supplementary Figure 5.- Characterisation of cells within activated cluster 1. (A)** UMAP visualisation of single cell RNA transcriptomes obtained from product samples within cluster 1 (refers to Figures 2A and 4A). The cells were coloured according to the expression levels of *CD4*. Colour scheme is based on ln scale of normalised counts from 0 (grey) to the indicated maximum value in the scale (dark red). **(B)** Subclusters for cells within cluster 1 were identified using the graph-base method Leiden. UMAP in A) was coloured according to the defined five subclusters. **(C)** Cells within subcluster 1-1 were compared to cells in the rest of subclusters of cluster 1. MA plot shows the results of the comparison as mean expression for each gene in the x-axis (expressed as log 2) and fold change in subcluster 1-1 when compared to the rest (expressed as log2) in the y-axis. Genes with adjusted p-value < 0.05, log2 fold change > |1| and mean expression >-5 are depicted in red. **(D)** Cells in UMAP visualisation in A) were coloured according to their condition. **(E)** Cells in UMAP visualisation in A) were coloured according to the expression levels of the CAR. Colour scheme is based on ln scale of counts from 0 (grey) to the indicated maximum value in the scale (dark red).

**Supplementary Figure 6.- Exhaustion markers distribution and donor contribution to exhausted cells. (A)** Gating strategy for results shown in Figure 5A. Plots correspond to the unstimulated sample from donor 1. RQR8 gating was based on an untransduced control and LAG3/ PD1/TIM3 gating was based on isotype controls. **(B)** Distribution of exhaustion score in all cells of the product samples from 3 donors. Cells above an exhaustion score of 0.6 (depicted) were considered as exhausted. **(C)** UMAP visualisation of single cell RNA transcriptomes obtained from product samples within cluster 1 (refers to Figures 2A and 4A). The cells were coloured according to their predicted exhaustion score. Colour scheme is based on the scale of predicted exhausted score from the lowest (grey) to the maximum score value in the scale (dark red). **(D)** UMAP visualisation of single cell RNA transcriptomes obtained using all product cells from 3 donors. The cells were coloured according to the expression levels of denoted genes related with exhaustion signature (*LAG3*, *HAVCR2*, *CTLA4*, *TIGIT* and *TBX21*). Colour scheme is based on ln scale of normalised counts from 0 (grey) to the indicated maximum value in each scale (dark red). **(E)** Pie chart representing the proportion of predicted exhausted cells from each donor.
