# Supplementary figures and images for: Single-cell transcriptome analysis of CAR T-cell products reveals subpopulations, stimulation, and exhaustion signatures
Source: Oncoimmunology. 2021 Jan 6;10(1):1866287. doi: 10.1080/2162402X.2020.1866287 (PMC7801130; doi:10.1080/2162402X.2020.1866287)

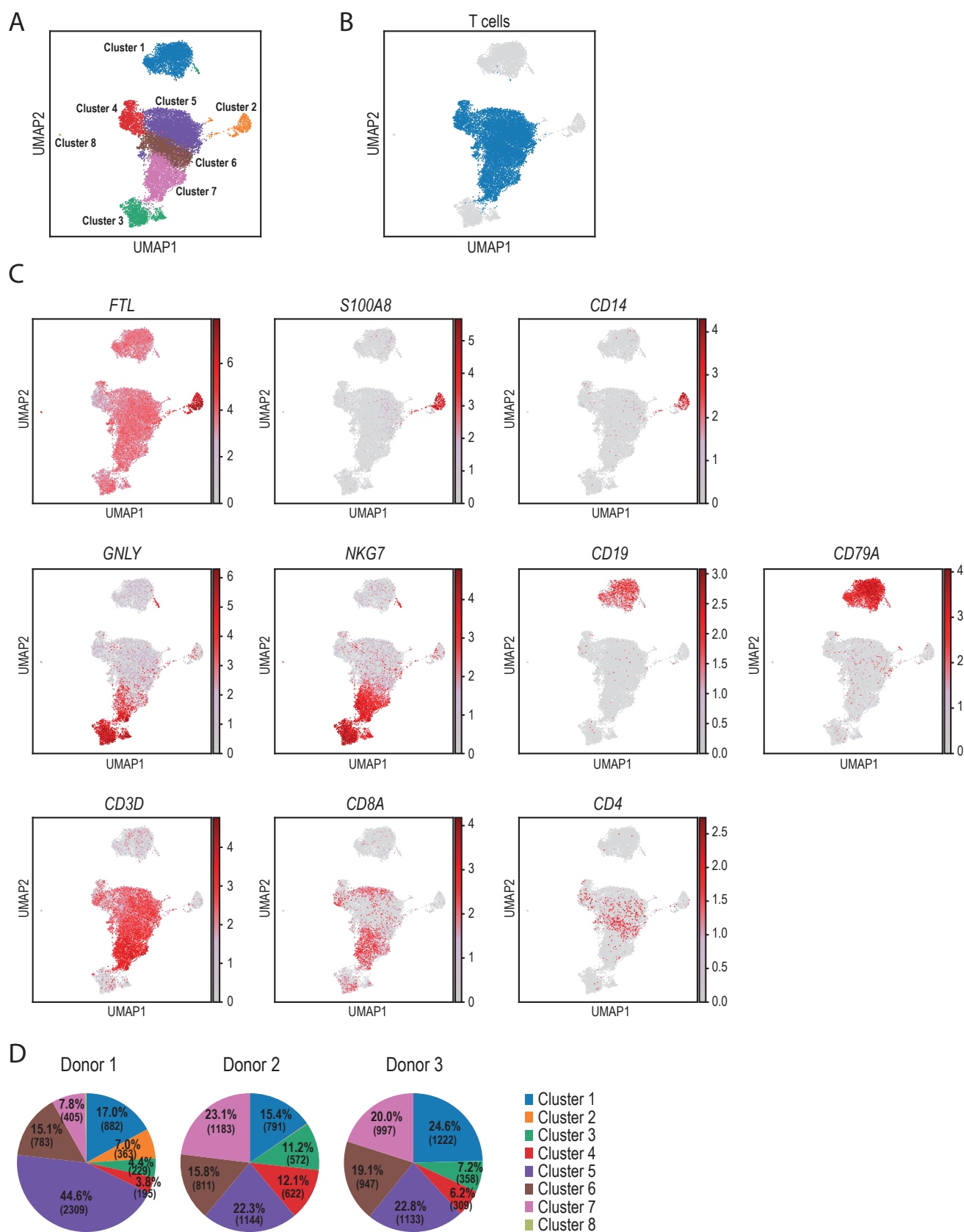

Wang et al. Extended Results Figure 1

Supplement: Supplemental Material [file KONI_A_1866287_SM5890.zip › supplementary figures/Wang_Extended Results Figure 1.pdf]

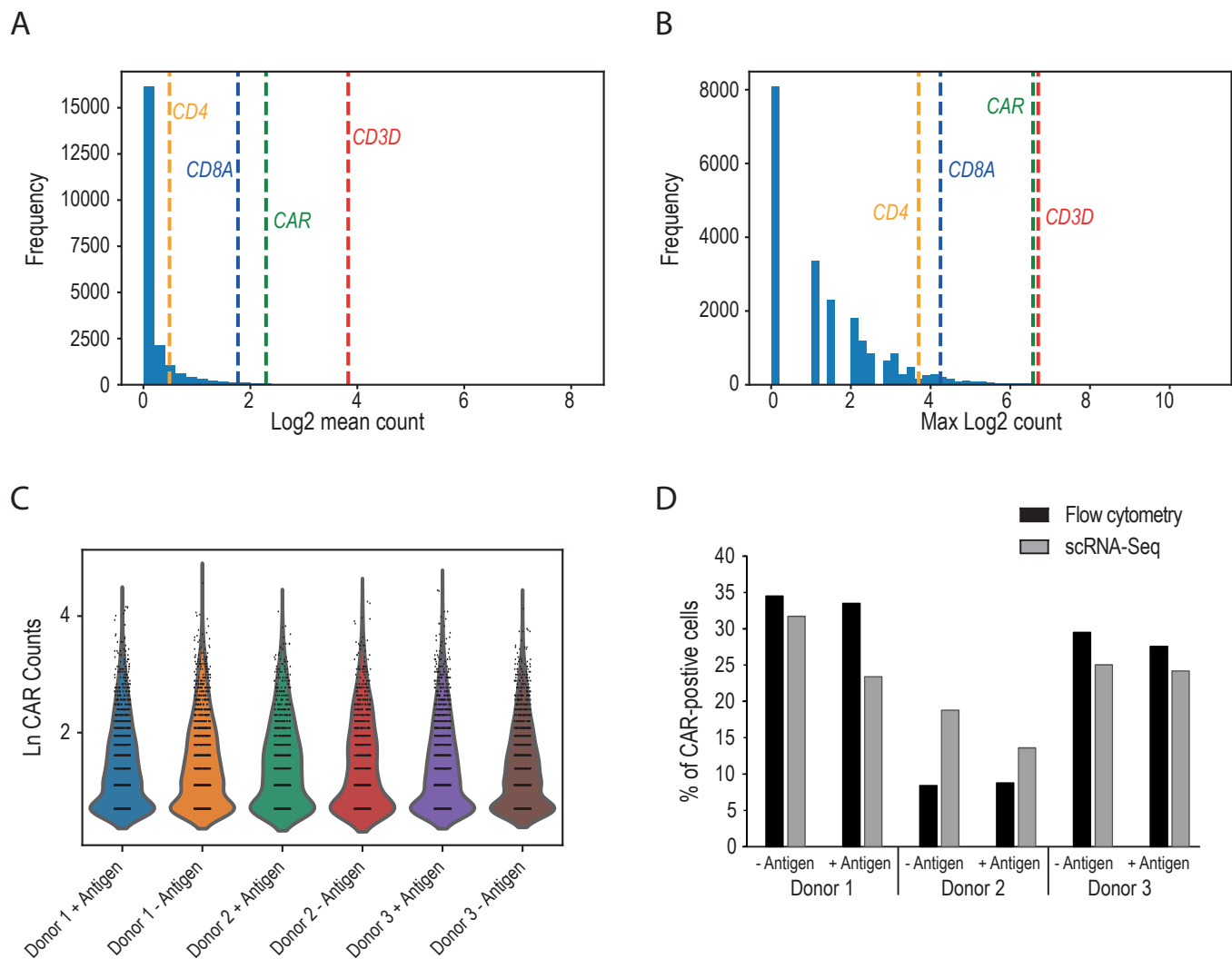

Wang et al. Supplementary Figure 1

Supplement: Supplemental Material [file KONI_A_1866287_SM5890.zip › supplementary figures/Wang_Suppl Figure 1_Resubmission.pdf]

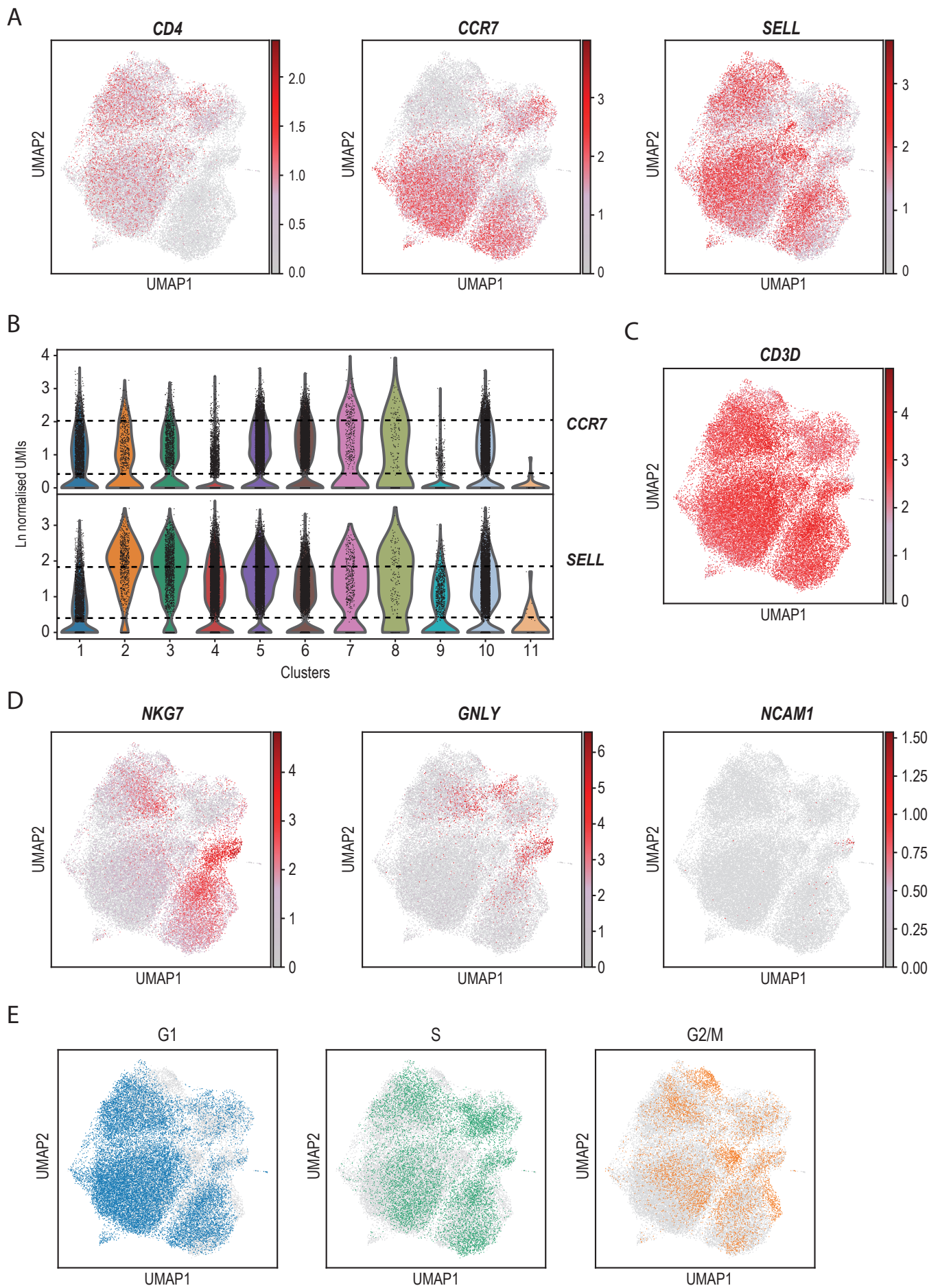

Wang et al. Supplementary Figure 2

Supplement: Supplemental Material [file KONI_A_1866287_SM5890.zip › supplementary figures/Wang_Suppl Figure 2_Resubmission.pdf]

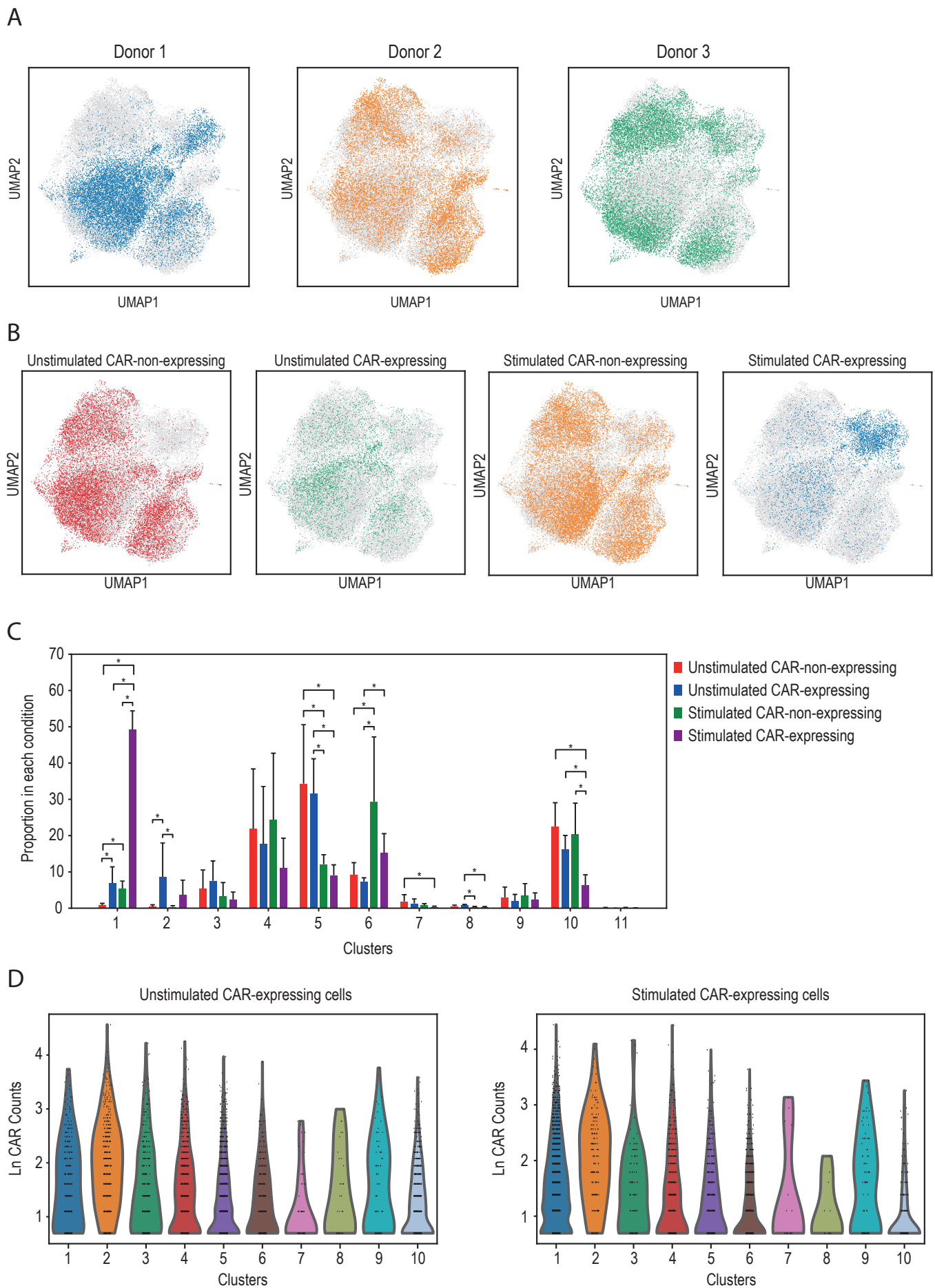

Wang et al. Supplementary Figure 3

Supplement: Supplemental Material [file KONI_A_1866287_SM5890.zip › supplementary figures/Wang_Suppl Figure 3_Resubmission.pdf]

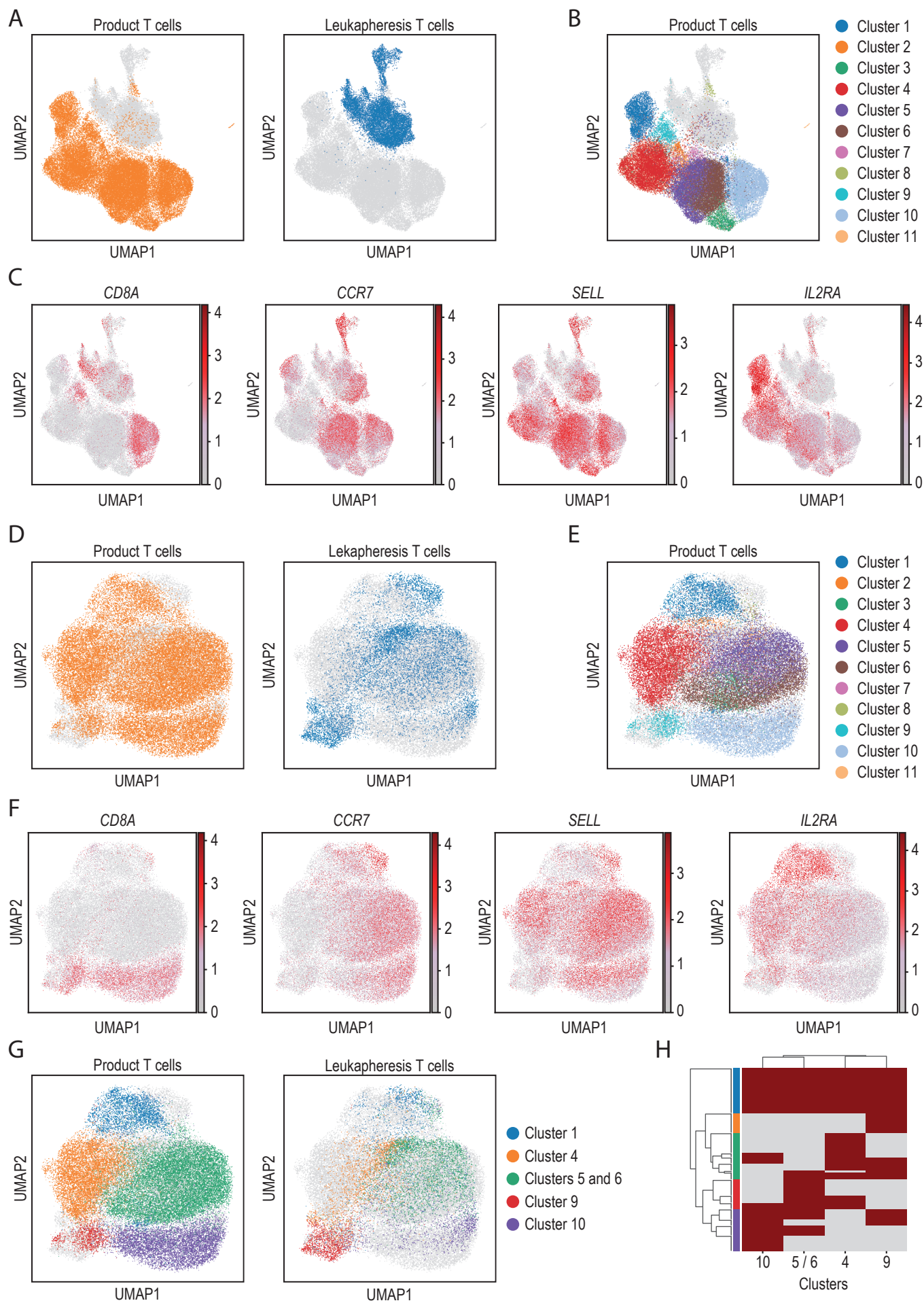

Wang et al. Supplementary Figure 4

Supplement: Supplemental Material [file KONI_A_1866287_SM5890.zip › supplementary figures/Wang_Suppl Figure 4_Resubmission_Rasterized.pdf]

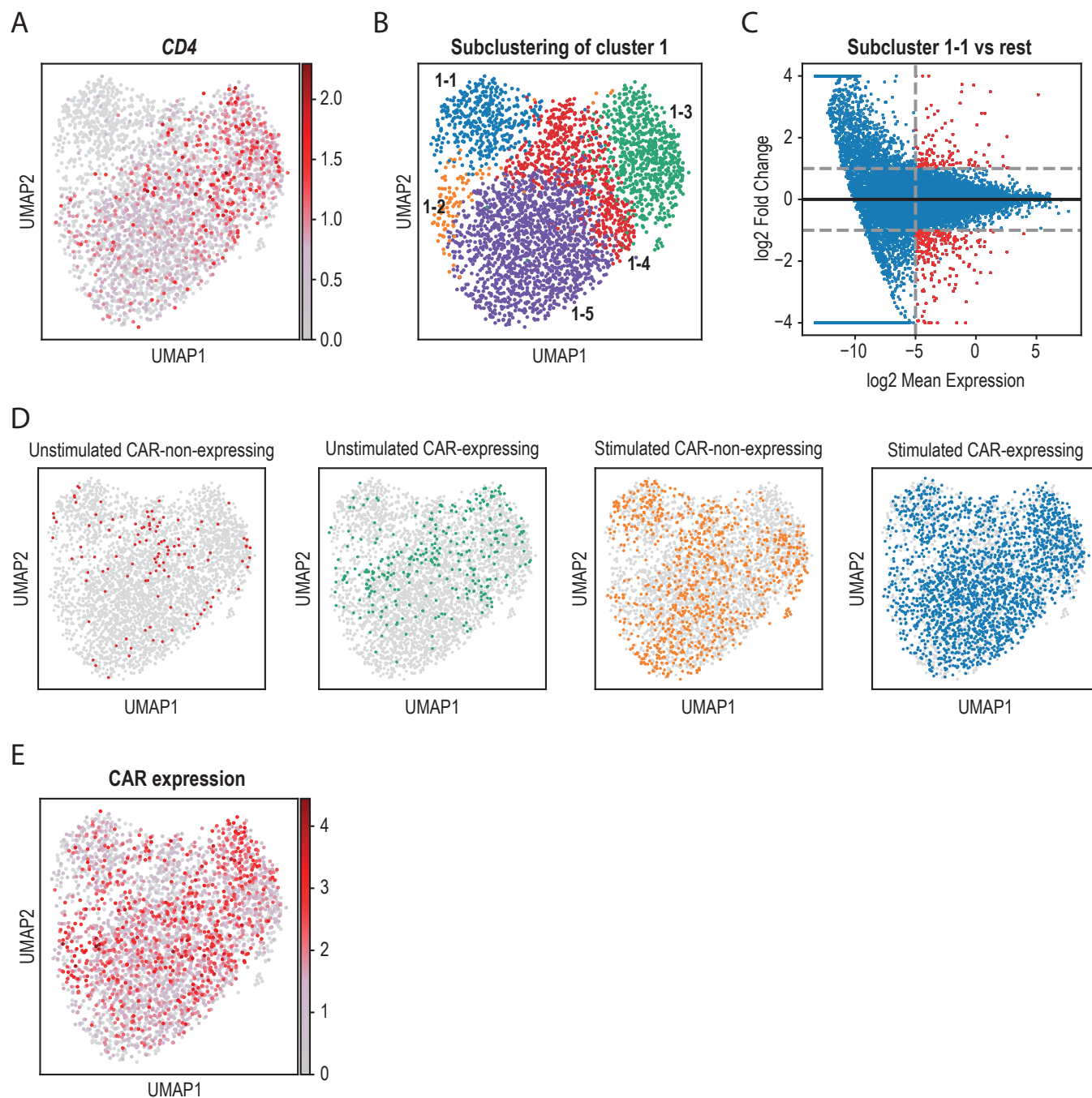

Supplement: Supplemental Material [file KONI_A_1866287_SM5890.zip › supplementary figures/Wang_Suppl Figure 5_Resumision.pdf]

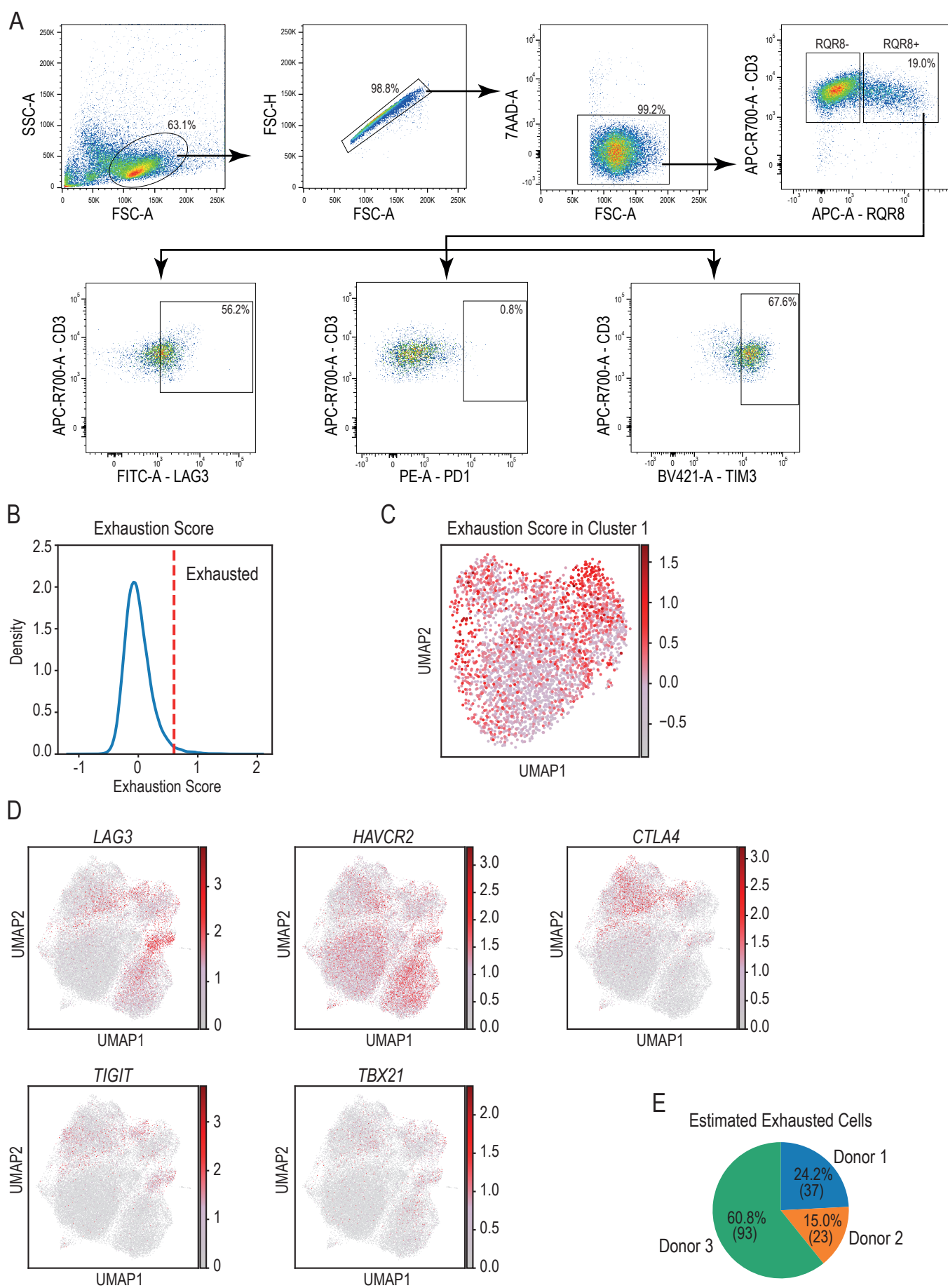

Wang et al. Supplementary Figure 6

Supplement: Supplemental Material [file KONI_A_1866287_SM5890.zip › supplementary figures/Wang_Suppl Figure 6_Resubmission2.pdf]
