# Supplementary material for: Single-cell transcriptome analysis of CAR T-cell products reveals subpopulations, stimulation, and exhaustion signatures
Source: Oncoimmunology. 2021 Jan 6;10(1):1866287. doi: 10.1080/2162402X.2020.1866287 (PMC7801130; doi:10.1080/2162402X.2020.1866287)
Supplement: Supplemental Material [file KONI_A_1866287_SM5833.zip › supplementary table/Description Supplementary Tables_Resubmission1.docx]

**Table 1.-** Distribution of samples in the study. This table shows the number of cells analysed for each condition (unstimulated CAR-non-expressing, unstimulated CAR-expressing, stimulated CAR-non-expressing, stimulated CAR-expressing) and their distribution into the different donors and clusters.

**Supplementary Table 1.-** Upregulated genes in T cells from the product compared to T cells from the leukapheresis. This table contains the upregulated genes in unstimulated CAR-non-expressing T cells from the different product samples when compared to leukapheresis T-cells. Comparisons were performed for cells associated to clusters 4, 5/6, 9 and 10. Genes that are contained within each of the different gene clusters defined in Supplementary Figure 4H are also listed.

**Supplementary Table 2.-** Differentially expressed genes in unstimulated CAR-expressing cells compared to unstimulated CAR-non-expressing cells. This table contains the output results of differential expression analysis when comparing unstimulated CAR-expressing cells and unstimulated CAR-non-expressing cells. Comparisons were performed for each cluster.

**Supplementary Table 3.-** Differentially expressed genes in cells within cluster 5 compared to cells within cluster 6. This table contains the output results of differential expression analysis when comparing cluster 5 and cluster 6 cells.

**Supplementary Table 4.-** Differentially expressed genes in cells within subcluster 1 compared to the rest of cells within cluster 1. This table contains the output results of differential expression analysis when comparing subcluster 1 and the rest of cells within the cluster 1.

**Supplementary Table 5.-** Differentially expressed genes in the comparison of stimulated and unstimulated CAR-expressing cells. This table contains the output results of differential expression analysis when comparing stimulated and unstimulated CAR-expressing cells within each cluster other than cluster 1.

**Supplementary Table 6.-** Differentially expressed genes in stimulated CAR-expressing cells within cluster 1 and stimulated CAR-expressing cells within the rest of clusters. This table contains the output results of differential expression analysis when comparing stimulated CAR-expressing cells within cluster 1 and stimulated CAR-expressing cells within the rest of clusters.

**Supplementary Table 7.-** Exhaustion score and signatures for GSEA. Lists of genes used for calculating the exhaustion score and for GSEA analysis in Figures 5E and 5F.

**Supplementary Table 8.-** Gene signature of exhausted CAR-expressing antigen-exposed cells. Output results of differential expression analysis when comparing the 153 CAR-expressing antigen-exposed cells with highest exhaustion score within cluster 1 with the 153 CAR-expressing antigen-exposed cells with lowest exhaustion score within the same cluster.
